# Viral infection triggers interferon-induced expulsion of live *Cryptococcus neoformans* by macrophages

**DOI:** 10.1371/journal.ppat.1008240

**Published:** 2020-02-27

**Authors:** Paula I. Seoane, Leanne M. Taylor-Smith, David Stirling, Lucy C. K. Bell, Mahdad Noursadeghi, Dalan Bailey, Robin C. May

**Affiliations:** 1 Institute of Microbiology & Infection and School of Biosciences, University of Birmingham, Edgbaston, Birmingham, United Kingdom; 2 Division of Infection and Immunity, University College London, London, United Kingdom; 3 The Pirbright Institute, Surrey, United Kingdom; Ohio State University, UNITED STATES

## Abstract

*Cryptococcus neoformans* is an opportunistic human pathogen, which causes serious disease in immunocompromised hosts. Infection with this pathogen is particularly relevant in HIV^+^ patients, where it leads to around 200,000 deaths *per annum*. A key feature of cryptococcal pathogenesis is the ability of the fungus to survive and replicate within the phagosome of macrophages, as well as its ability to be expelled from host cells via a novel non-lytic mechanism known as vomocytosis. Here we show that cryptococcal vomocytosis from macrophages is strongly enhanced by viral coinfection, without altering phagocytosis or intracellular proliferation of the fungus. This effect occurs with distinct, unrelated human viral pathogens and is recapitulated when macrophages are stimulated with the anti-viral cytokines interferon alpha or beta (IFNα or IFNβ). Importantly, the effect is abrogated when type-I interferon signalling is blocked, thus underscoring the importance of type-I interferons in this phenomenon. Lastly, our data help resolve previous, contradictory animal studies on the impact of type I interferons on cryptococcal pathogenesis and suggest that secondary viral stimuli may alter patterns of cryptococcal dissemination in the host.

## Author summary

Infectious diseases are typically studied in isolation, but in real life people often encounter multiple infections simultaneously. Here we investigate how the innate immune response to the fatal fungus *Cryptococcus neoformans* is influenced by viral coinfection. Whilst virally-infected macrophages retain a normal capacity to engulf and kill Cryptococci, they demonstrate a dramatically enhanced propensity to expel them through vomocytosis. Activation of vomocytosis is driven by type-I interferons, generic ‘antiviral’ molecules, which signal back to the infected macrophage, triggering expulsion of the fungus. We propose that this hitherto unobserved phenomenon represents a ‘reprioritisation’ pathway for innate immune cells, by which they can alter the frequency with which they expel one pathogen depending on the level of threat from a secondary infection.

## Introduction

Since their discovery in 1957 by Isaacs and Lindenmann [1], the antiviral effects of type I interferons have been well documented [2–4]. More recently, their roles in non-viral infections have been investigated [5, 6]. Different bacterial stimuli have been shown to elicit type I interferon production, and in turn these so-called “antiviral cytokines” play a role in the outcome of bacterial infections [7–9]. This stems in part from the complex and sometimes contradictory effects that type I interferons have on host cells, for instance in enhancing inflammatory responses in some infectious settings [6] to preventing hyperinflammation in others [10, 11], and even affecting the priming of immune responses at lymph nodes [12].

To date, little is known about the interplay between type I interferons and fungal infections, despite the fact that many life-threatening fungal infections occur in the context of chronic viral infection. This is particularly true of *Cryptococcus neoformans*, a globally distributed opportunistic pathogen that is responsible for nearly 200,000 deaths per year in human immunodeficiency virus (HIV) infected patients, where it causes cryptococcal meningitis [13]. Extensive work over many years has demonstrated that a key feature of cryptococcal pathogenesis is the ability of the fungus to survive, proliferate within, and then escape from, host macrophages [14–17]. Macrophages are among the first immune cells to encounter the fungus within the human host [18], and thus are very important in the fight against this pathogen. These cells are able to phagocytose and contain the threat, as happens in immunocompetent hosts, but can also by hijacked by Cryptococcal cells and used as a “Trojan horse” to disseminate to distal sites within the body, particularly to the central nervous system [19]. Engulfed Cryptococcal cells can escape from host macrophages through lytic or non-lytic mechanisms, the latter being known as vomocytosis or non-lytic extrusion [20, 21]. Most studies to date have focused on the interaction of *Cryptococcus* with healthy host cells, and consequently the impact of viral coinfection on this intracellular lifestyle remains largely unknown.

Here we show that viral infections enhance vomocytosis of Cryptococci from infected macrophages, without affecting phagocytosis or intracellular proliferation rate of the fungus. This effect is lost when signalling through the type I interferon receptor is blocked, and can be recapitulated by addition of exogenous IFNα or IFNβ. Re-interpreting previous data from murine cryptococcosis models suggests that interferon-mediated enhancement of vomocytosis may help protect against CNS dissemination early during an infection, but accelerate pathogenesis at later time points [22]. Together these findings reveal a hitherto unknown facet of the host response to systemic fungi and suggest that secondary viral exposure may be an important modulator of disease progression during cryptococcosis.

## Results

**Fig 1:**
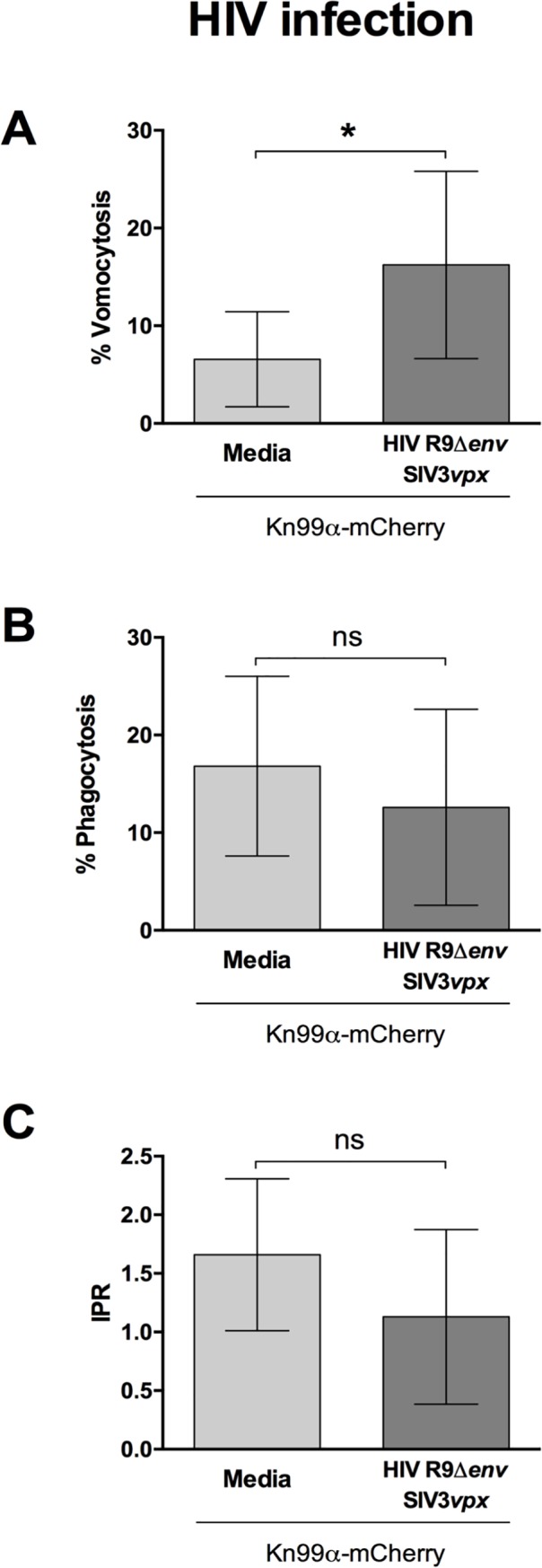
HIV infection enhances vomocytosis of *C*. *neoformans*. Human monocyte-derived macrophages were infected with HIV and subsequently infected with *C*. *neoformans*. Time-lapse microscopy videos were manually scored for vomocytosis (top), uptake (middle) and intracellular proliferation rate of *C*. *neoformans* (bottom). **A** Graph shows percentage of *Cryptococcus*-infected macrophages which have experienced at least one vomocytosis event. **B** Percentage of *Cryptococcus*-infected macrophages. **C** Intracellular proliferation rate of *C*. *neoformans* over 18 hours. In all cases, pooled data from 9 independent experiments is shown. Categorical vomocytosis and phagocytosis data was analysed by Chi^2^ test followed by Fisher's exact test. * p < 0.05. IPR data was analysed using Mann-Whitney test.

Given the relevance of cryptococcosis to HIV^+^ patients [13], we initially set out to test whether HIV infection had an effect on vomocytosis of *C*. *neoformans*. Human monocyte-derived macrophages were infected with HIV-1 capable of a single-round of infection and subsequently with *C*. *neoformans* and then used for time-lapse imaging over 18 hours. Subsequent scoring showed that virally infected cells had a significantly higher occurrence of cryptococcal vomocytosis (Fig 1A), whilst fungal uptake and intracellular proliferation were unaltered (Fig 1B and 1C).

The experimental HIV system we used here includes co-transduction with SIV3*vpx* VLPs in order to counteract the antiviral effect of SAMHD1 and ensure maximal HIV infection of the macrophages [23, 24] (S1A Fig). Interestingly, we noted that the addition of SIV3*vpx* or R9HIVΔ*env* alone also increased vomocytosis (S1B Fig). Since neither condition results in widespread viral infection of host cells, this suggested that the enhancement of vomocytosis occurs at the level of viral detection, rather than being a consequence of active HIV infection.

**Fig 2:**
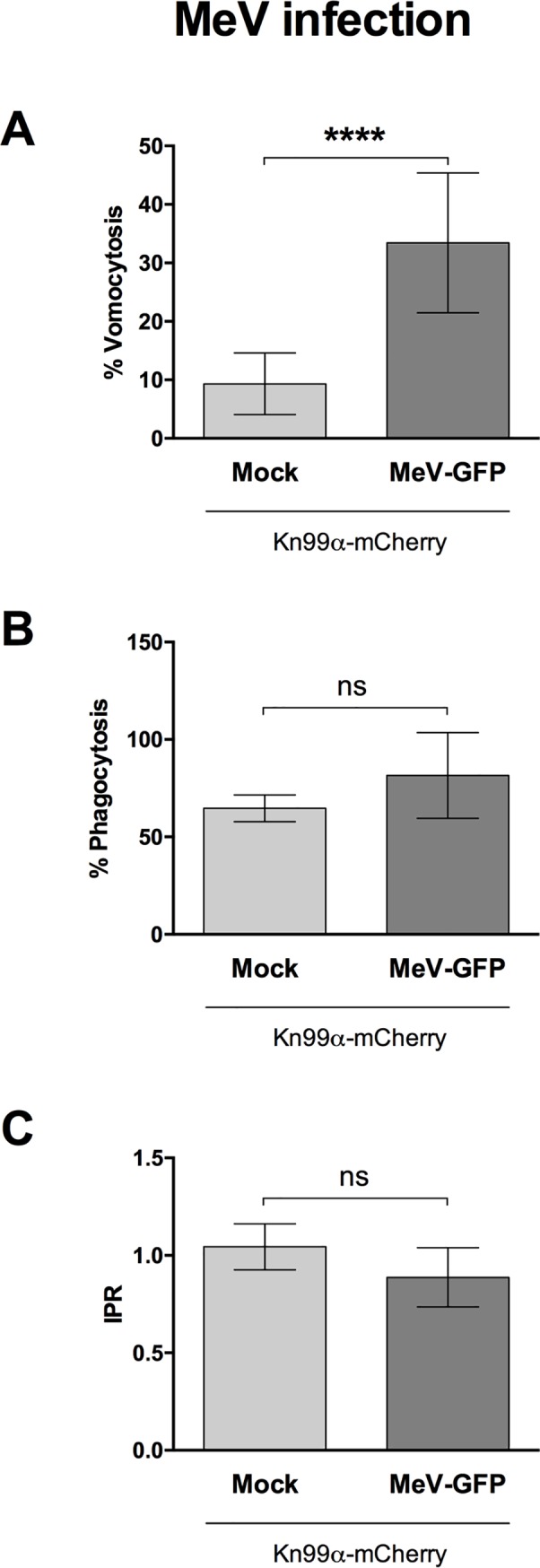
Measles infection enhances vomocytosis of *C*. *neoformans*. Human monocyte-derived macrophages were infected with Measles virus and subsequently infected with *C*. *neoformans*. Time-lapse microscopy videos were manually scored for vomocytosis (top), uptake (middle) and intracellular proliferation rate of *C*. *neoformans* (bottom). **A** Graph shows percentage of *Cryptococcus*-infected macrophages which have experienced at least one vomocytosis event. **B** Percentage of *Cryptococcus*-infected macrophages. **C** Intracellular proliferation rate of *C*. *neoformans* over 18 hours. In all cases, pooled data from 3 independent experiments is shown. Categorical vomocytosis and phagocytosis data was analysed by Chi^2^ test followed by Fisher's exact test. **** p < 0.0001. IPR data was analysed using Mann-Whitney test.

To explore this further, we tested whether vomocytosis was altered in macrophages infected with an unrelated macrophage-tropic virus [25]; measles (MeV, Fig 2A). The measles strain used represents a virulent field isolate from Japan. Once again, infection with the virus resulted in significantly enhanced vomocytosis of *Cryptococcus*. Neither HIV nor measles infection affected uptake of *Cryptococcus* nor the intracellular proliferation rate (IPR) of the fungus (Fig 1B and 1C and Fig 2B and 2C), suggesting that the viral effect acts specifically at the level of vomocytosis, rather than fungal pathogenicity *per se*, and that it is independent of the type of virus.

**Fig 3:**
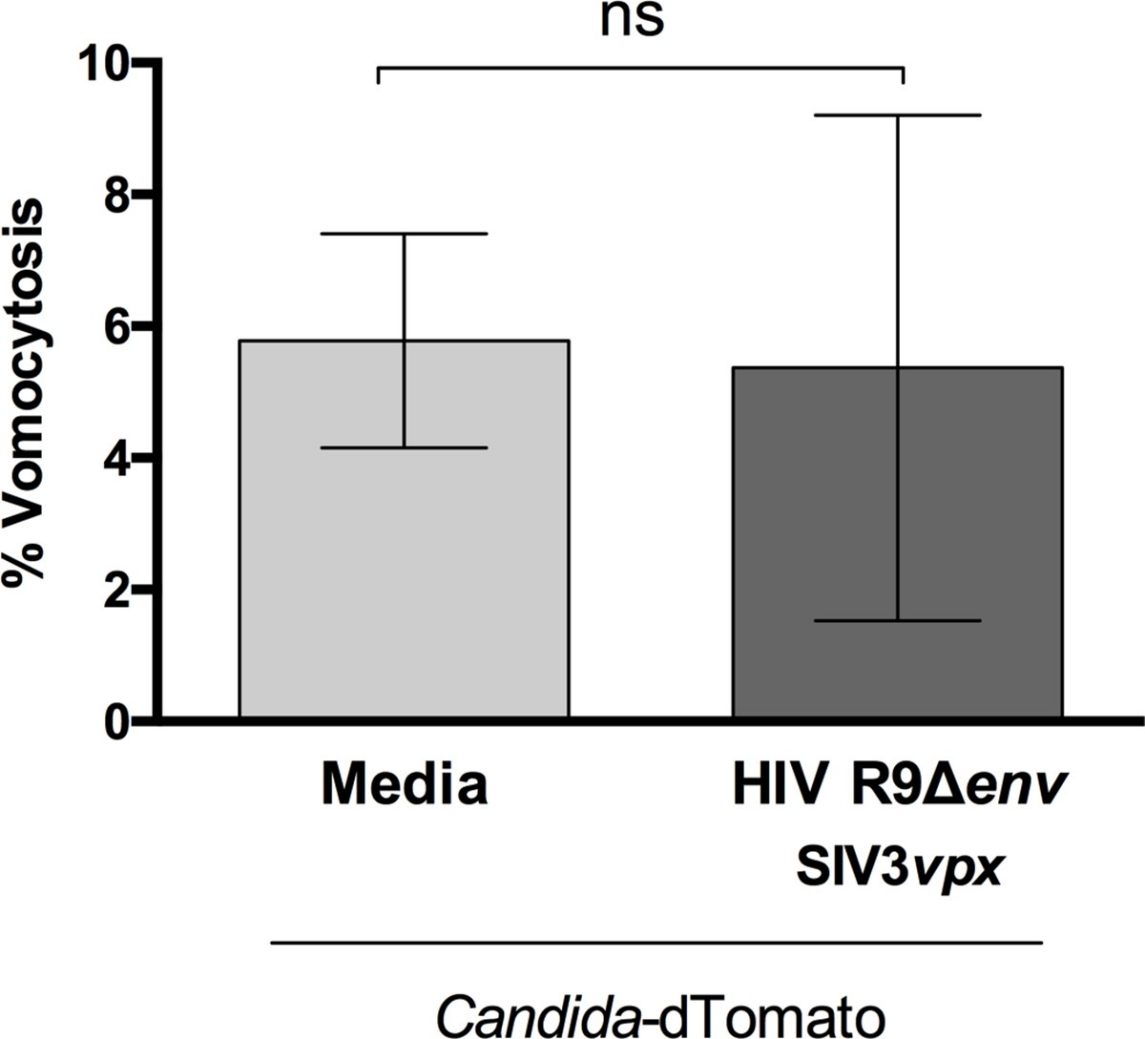
Viral enhancement of vomocytosis does not occur with *Candida albicans*. Human monocyte-derived macrophages were infected with HIV and subsequently infected with fluorescently-labelled *C*. *albicans*. Time-lapse microscopy videos were manually scored for vomocytosis. Graph shows Mean + SD of percentage of *Candida*-infected macrophages which have experienced at least one vomocytosis event. Chi^2^ test followed by Fisher's exact test performed on raw vomocytosis counts. Pooled data from two independent experiments.

Vomocytosis has been reported in *Cryptococcus* infection [20, 21], but also in *Candida krusei* [26] and *Candida albicans* [27]. To assess whether the effect seen is *Cryptococcus*-specific, we infected human monocyte-derived macrophages with HIV-1 in the presence of vpx-containing VLP, as before, and subsequently infected with a strain of *Candida albicans* expressing dTomato and over-expressing the protein NRG1. NRG1 over-expression inhibits filamentation in *Candida* [28], thus preventing the fungus from forming hyphae which lyse the host-macrophage prior to vomocytosis. Time-lapse imaging of this *Candida*:HIV1 co-infection did not show the same increased level of vomocytosis observed with *Cryptococcus* (Fig 3), suggesting that viral-induced expulsion may not be common to all intracellular fungal pathogens.

**Fig 4:**
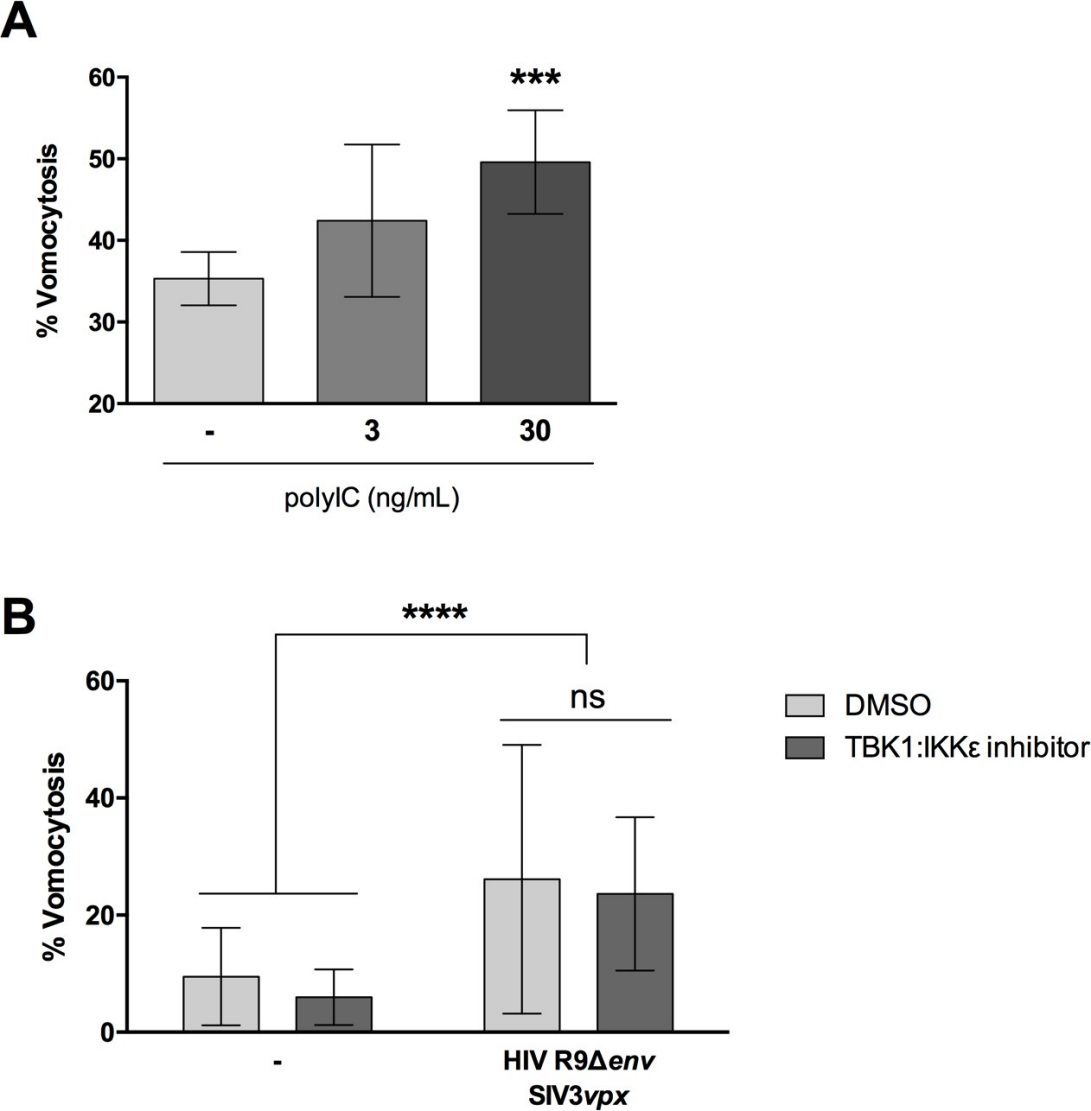
Viral enhancement of vomocytosis is not TBK1:IKKε dependent. Human monocyte-derived macrophages were stimulated with different doses of polyIC (**A**) or infected with attenuated HIV (**B**), and subsequently infected with *C*. *neoformans*. In **B**, infection was carried out in the presence or absence of TBK1:IKKε inhibitor (dark gray). Time-lapse microscopy videos were manually scored for vomocytosis. Graphs show Mean + SD of percentage of *Cryptococcus*-infected macrophages which have experienced at least one vomocytosis event. Chi^2^ test followed by Fisher's exact test performed on raw vomocytosis counts. Pooled data from at least three independent experiments.

To test whether active viral infection was required for enhanced vomocytosis of *C*. *neoformans*, we mimicked the effect of viral exposure by stimulating macrophages with polyinosinic-polycytidilic acid (polyIC). PolyIC is a double-stranded RNA synthetic analogue, which is known to trigger antiviral responses by binding to TLR3 [29]. Human monocyte-derived macrophages were stimulated with polyIC, infected with *C*. *neoformans* simultaneously and then scored for vomocytosis. As with HIV or MeV infection, polyIC stimulation showed a dose-dependent enhancement of vomocytosis, although to a lesser extent than exposure to complete virus (Fig 4A).

To investigate potential mechanisms by which this enhancement of vomocytosis may be accomplished, we tested a TBK1:IKKε inhibitor in the presence of a viral infection (Fig 4B). TBK1:IKKε is a downstream signalling molecule that is engaged during viral sensing and plays a key role in regulating the ensuing antiviral response. Interestingly, the inhibitor was capable of limiting the effects of polyIC stimulation on vomocytosis, as well as blocking polyIC-induction of IFNα (S2 Fig), but did not block the enhanced vomocytosis seen when macrophages are exposed to complete virus (Fig 4B). Thus, it is likely that the systemic antiviral reaction of the host macrophage, rather than a single anti-viral signalling pathway, is the trigger for enhanced vomocytosis from infected host cells.

**Fig 5:**
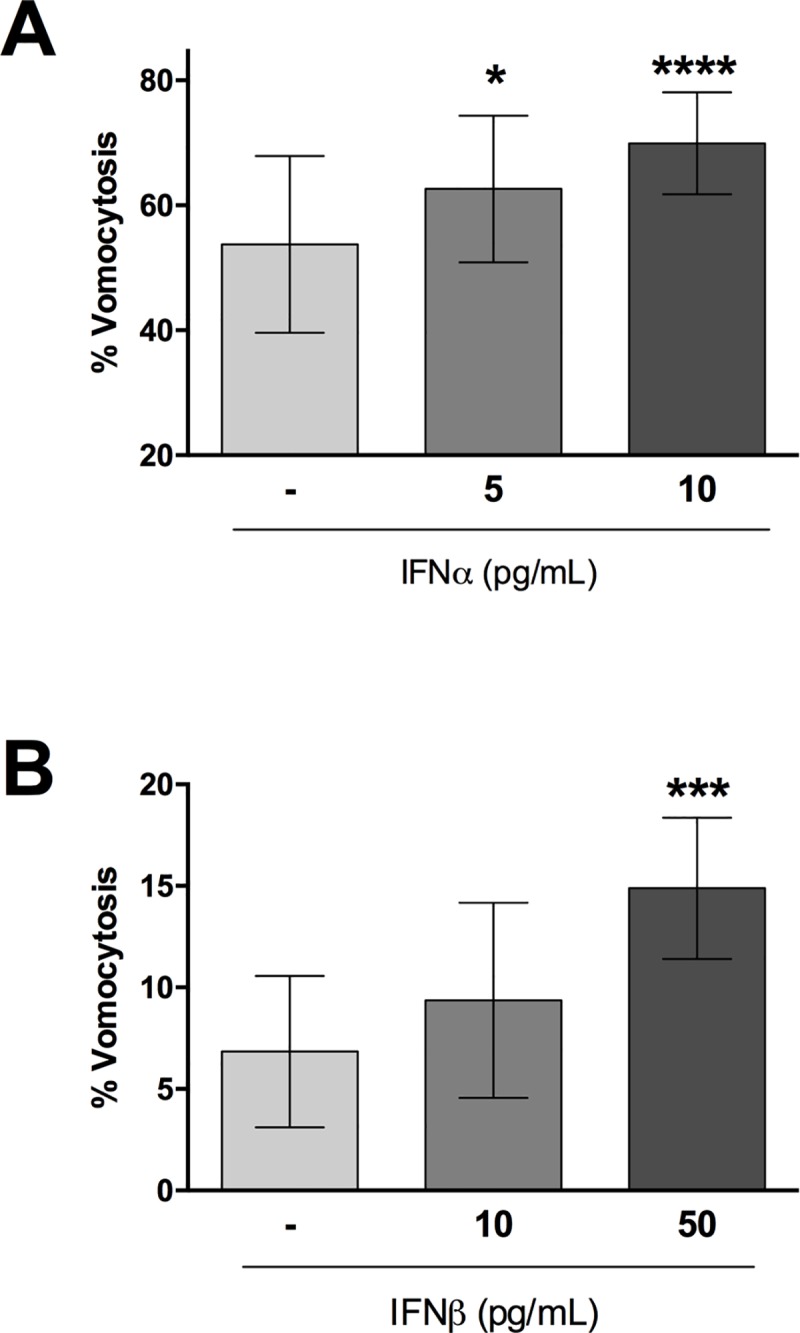
Antiviral response increases vomocytosis. Human monocyte-derived macrophages were stimulated with different doses of IFNα (**A**) or IFNβ (**B**), and infected with *C*. *neoformans*. Graphs show Mean + SD of percentage of *Cryptococcus*-infected macrophages which have experienced at least one vomocytosis event. Chi^2^ test followed by Fisher's exact test performed on raw vomocytosis counts. Pooled data from at least three independent experiments.

The hallmark of the cellular anti-viral response is the induction of type-I interferons. Among these, the best studied are IFNα and IFNβ. During HIV infection specifically, the induction of IFNα is the most relevant [30]. We therefore tested whether the impact of viral infection on vomocytosis could be recapitulated by exposure to IFNα or IFNβ. Stimulation of human monocyte-derived macrophages with 10 pg/mL IFNα (a level that closely matches that seen in HIV-infected patients [30]) resulted in significantly enhanced vomocytosis of *Cryptococcus* (Fig 5A) without altering cryptococcal growth, uptake or IPR (S3 Fig). The same effect could be seen when stimulating with 50 pg/mL IFNβ (Figs 5B and S3). Interestingly, we noticed that higher doses of interferon suppressed this effect, suggesting that the impact of interferons on vomocytosis can be rapidly saturated.

**Fig 6:**
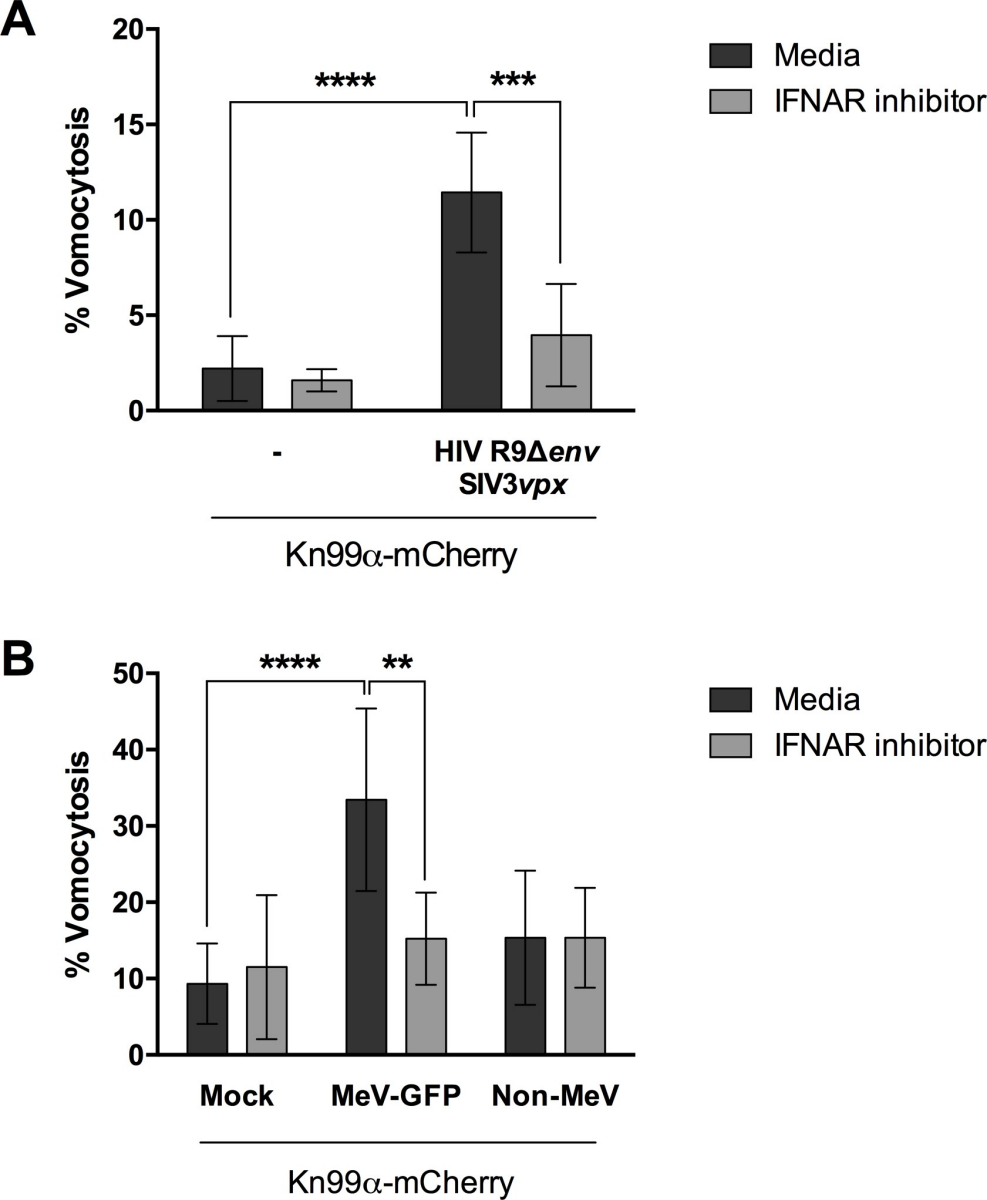
Type-I interferon signalling is necessary to enhance vomocytosis. Human monocyte-derived macrophages were infected with HIV (**A**) or GFP-expressing measles virus (MeV-GFP, **B**) and subsequently with mCherry-expressing *C*. *neoformans* (Kn99α-mCherry), in the presence or absence of an IFNAR blocking antibody. GFP negative cells, which did not have an active Measles infection, were termed “Non-MeV”. Graph shows Mean + SD of percentage of *Cryptococcus*-infected macrophages which have experienced at least one vomocytosis event. Fisher's exact test performed on raw vomocytosis counts. Pooled data from two and three biological repeats, respectively.

To confirm that type-I interferons were behind the increase in vomocytosis observed, we performed the viral infection experiments in the presence of a type-I interferon receptor (IFNAR) inhibitor (Fig 6). The addition of IFNAR inhibitor blocked the enhancement of vomocytosis otherwise elicited by viral infection in both HIV- and Measles-infection settings, confirming that type-I interferon signalling is necessary for this effect. Interestingly, this effect was particularly prominent on virally infected cells rather than non-infected neighbouring cells (Non-MeV; Fig 6B), suggesting that the impact of type-I interferon signalling on vomocytosis is highly localised and specific to the autocrine responses occurring within infected cells, rather than endocrine responses mediated through other cytokines.

## Discussion

In this study we set out to explore the consequences of viral infection on Cryptococcal infection, focusing on the non-lytic escape mechanism known as vomocytosis. Infection with either HIV or measles virus led to an enhancement in vomocytosis of *C*. *neoformans*, without affecting uptake or intracellular proliferation of the fungus (Figs 1 and 2). The effect on *Cryptococcus* was not impaired when specifically blocking TBK1:IKKε activation (Fig 4) but could be recapitulated by stimulation with IFNα or IFNβ and abrogated when signalling from type-I interferon receptor was blocked (Figs 5 and 6). Thus, viral coinfection stimulates expulsion of intracellular Cryptococci via Type I interferon signalling. Interestingly, viral infection did not affect vomocytosis of *Candida albicans* (Fig 3), raising the possibility that viral-induced expulsion may be pathogen-dependent. However, we note that intracellular infection by *Candida* differs in many respects from that with *Cryptococcus* (e.g. the occurrence of filamentation, the cell surface molecules expressed and the overall burden of infection) and consequently a more detailed understanding of this difference awaits future investigation.

The effect was seen using two distinct viral pathogens which differ, among other parameters, in the magnitude of the anti-viral response they elicit in human macrophages. Relative to other viral infections, HIV is very good at avoiding the induction of type-I interferons [31, 32]. Nonetheless, the low levels of type-I interferons induced by HIV, potentially enhanced by the co-infection with *Cryptococcus*, are sufficient to have a significant effect on vomocytosis. Importantly, our data indicate that viral detection, rather than productive infection, is the critical trigger for enhanced vomocytosis. Consequently, even transient exposure to viral threats may alter the outcome of *Cryptococcus*/macrophage interactions, even for pathogens (such as measles) for which there is no apparent disease association with cryptococcosis.

Why might antiviral signalling induce vomocytosis? One possibility is that vomocytosis serves to “reset” phagocytes that have been unable to kill their prey, thus allowing them to serve a useful purpose in phagocytosing other pathogens rather than remaining “unavailable”. In that context, a potent inflammatory signal such as IFNα/β may serve to accelerate this process during localised infection, returning macrophages to functionality faster than would otherwise occur.

One consequence of this is that vomocytosis may either inhibit or accelerate disease progression, depending on the timing and context of the event. For instance, cryptococcal vomocytosis within the lung at an early stage of infection might be beneficial, since it would limit the risk of ‘Trojan Horse’ dissemination of the organism to other tissues. In contrast, once the fungus has reached the blood stream or CNS, enhanced vomocytosis is likely to be deleterious, since it would allow the pathogen to escape the constraints of a phagocyte and grow rapidly in the extracellular environment.

Indeed the former hypothesis is strongly supported by *in vivo* data from several groups. Sionov *et al* [22] showed that stimulation with IFNα or with the double-stranded RNA analogue pICLC protected the host from infection by either *C*. *neoformans* or *C*. *gatti* infection. This effect was time-dependent, with the protective effect of pICLC treatment only occurring if administered during the first 72 hpi, before the fungus reaches the brain. In contrast Sato *et al*. [33] showed that IFNARKO mice have a lower fungal burden than WT mice during advanced disease, whilst Oliveira *et al* [34] show that infection with influenza virus worsens the prognosis of *C*. *gattii* infection. These apparently conflicting data are all compatible with a model in which viral/interferon-driven stimulation of vomocytosis is protective during early, pulmonary infection by Cryptococci, but accelerates disease once the fungus has begun to disseminate.

Taken together, our findings therefore suggest that the antiviral response, and IFNα/β in particular, induce the expulsion of intracellular cryptococci and that this effect could be advantageous or detrimental to the host, depending on the localization of the infected phagocyte and timing of the event.

## Materials and methods

All reagents were purchased from SIGMA unless otherwise stated.

### Fungal strains

Cryptococcal strains were grown in Yeast Peptone Dextrose (YPD) broth [2% glucose, 1% peptone and 1% yeast extract] at 25°C on a rotator (20 rpm). Yeast from overnight cultures were centrifuged at 6500 rpm for 2 minutes and resuspended in PBS at the required concentration. All experiments were carried out using *C*. *neoformans var*. *grubii* serotype A strain Kn99α. Wildtype, GFP- [35] or mCherry-expressing [36] derivatives of Kn99α were used, as stated for each figure.

*Candida albicans*, expressing dTomato and over-expressing NRG1, was a kind gift from Dr. Robert Wheeler. It was grown in YPD broth at 37°C in a shaker incubator (200 rpm). Overnight cultures were washed with PBS and resuspended at the required concentration.

### Virus strains

HIV-1 virus stocks were generated by transfection of human embryonic kidney 293T cells (European Collection of Authenticated Cell Cultures) as previously described [31, 32]. The R9HIVΔ*env* virus was derived from clade B HIV-1 strain (NL43) with 500bp deletion in *env*, pseudotyped with vesiculostomatitis virus G envelope. SIV3mac single round virus like particles (VLPs) containing vpx (SIV3vpx) were generated by transfection into 293T cells with pSIV3+ and pMDG plasmids [23, 37]. At 48, 72h and 96h viral containing supernatant was harvested, centrifuged at 800 x g for 10 min and filtered through 0.45 um filter then centrifuged on a 20% sucrose cushion at 20,000 x g for 2h at 4°C. Purified virus was then re-suspended in RPMI media and frozen at -80°C. To quantify single round HIV infection, a vial was thawed for each harvest and serial dilutions used to infect CCR5/CD4 and CXCR4/CD4 transfected NP-2 cells. At 72h post infection wells were fixed in ice cold acetone-methanol and infected cells were identified by staining for p24 protein using a 1:1 mixture of the anti-p24 monoclonal antibodies EVA365 and EVA366 (NIBSC, Center for AIDS Reagents, UK). Infected cells were detected by light microscopy to provide a virus titre (focus-forming U/mL). The SIV3vpx particles were quantified after thawing using a reverse transcriptase (RT) assay colorimetric kit (Roche) following the manufacturer’s instructions to provide a RT ng/mL titre.

Recombinant MeV strain IC323 expressing green fluorescent protein [MeV-GFP] was generated as previously reported by Hashimoto *et al*. [38] MeV-GFP represents a virulent field isolate from Japan (Ichinose-B (IC-B) strain) and was isolated from a patient with acute measles in 1984 [39]. For the generation of virus stocks, Vero (ATCC CCL-81) cells overexpressing human SLAMF1 receptor (vero-hSLAM cells) were grown in T75 tissue culture flasks to approximately 80% confluency in DMEM supplemented with 0.4 mg/mL G418. Flasks were infected with MeV-GFP at an MOI of 0.01:1 in 5 mL media for 1 hour at 37°C. After 1h a further 10 mL of DMEM supplemented with 10% FBS was added and infection allowed to continue for 48 h. At harvest the flasks were frozen to -80°C. After thawing, the collected supernatants were centrifuged at 2500 rpm for 10 min at 4°C to pellet cell debris. Aliquoted virus in supernatant was then frozen to -80°C. MeV-GFP viruses were then titred using the TCID-50 method. Vero-hSLAM cells were seeded into flat-bottomed 96 well plates and infected with serial dilutions of thawed MeV-GFP in triplicate. After 72 h, wells were scored for positive or negative infection under UV illumination on a Nikon TE-2000 microscope.

### Ethics statement

All work with human tissue was approved by the University of Birmingham Ethics Committee under reference ERN_10–0660. Samples were collected specifically for this work and were not stored beyond the duration of the experiments described herein. All donors were adults and provided written informed consent prior to donation.

### Human macrophage isolation and culture

20–40 mL of blood were drawn from healthy donors by venepuncture. 6 mL of whole blood were carefully layered on top of a double layer of Percoll (densities of 1.079 and 1.098 g/mL). Samples were centrifuged in a swing bucket rotor at 150g for 8 minutes, followed by 10 minutes at 1200g, with acceleration and break set to zero. The resulting white disc of peripheral blood mononuclear cells (PBMC) was transferred to a clean vial and incubated with red blood cell lysis buffer at a ratio of 1:3 for 3 minutes, with gentle mixing throughout to prevent clot formation. Cells were then washed with ice cold PBS twice, with centrifugation at 400g for 6 minutes in between each wash, and counted with a haemocytometer. 1x10^6^ PBMC were seeded onto 48-well plates in RPMI-1640 media containing 1% penicillin/streptomycin, 5% heat-inactivated AB human serum and 20 ng/mL M-CSF (Invitrogen). Cells were washed with PBS and resuspended in fresh media on days 3 and 6 of differentiation. Macrophages were ready to use on day 7. A yield of 1x10^5^ macrophages per well was estimated.

### Fungal infection

Cryptococci were opsonised with 10% human AB serum or 18B7 antibody (a kind gift from Arturo Casadevall) for 1 hour and then added to macrophages at a multiplicity of infection (MOI) of 10:1. Infection was carried out in serum free-media, at 37°C with 5% CO_2_. After 2 hours, cells were washed 3 times with PBS to remove any extracellular fungi and fresh serum free-media was added.

In the case of *Candida* infections, fungal cells were added unopsonised to macrophages, at an MOI of 5:1. Infection was carried out in serum free-media, at 37°C with 5% CO_2_. After 45 minutes, cells were washed 3 times with PBS to remove any extracellular fungi and fresh serum free-media was added.

### Drug treatments

Exogenous compounds were added to macrophages at two stages; when infecting with *Cryptococcus* and again when replenishing with fresh media after removing extracellular fungi. Compounds tested include interferon alpha and beta (IFNα and IFNβ) at concentrations ranging from 5 to 100 pg/mL (Bio-Techne and RD Systems, respectively), polyinosinic-polycytidilic acid (polyIC) at 3 and 30 ng/mL (Invivogen), TBK1:IKKε inhibitor at 1 μM (BX795, Invivogen) and type-I interferon receptor inhibitor (IFNARinh) at 2.5 μg/mL (pbl assay science).

### Co-infection assay

Human monocyte-derived macrophages were infected with either attenuated human immunodeficiency virus (HIV) or MeV-GFP as follows:

For attenuated HIV co-infections, 24h before cryptococcal infection, human monocyte-derived macrophages were infected either with R9HIVΔ*env* at a MOI of 10:1, SIV3*vpx* at 3 ng/mL or both in serum free RPMI. At 24 h post infection duplicate wells were fixed in ice cold acetone-methanol and infected cells were identified by staining for p24 protein as described above. Experimental wells were infected with antibody opsonised-*Cryptococcus* Kn99α-GFP for 2 hours or unopsonised-*Candida* for 45 minutes, washed to remove extracellular fungal cells, and replenished with fresh serum free-media.

Alternatively, macrophages were infected with MeV-GFP at an MOI of 5:1 in serum free-media and kept at 37°C with 5% CO_2_. After 24 hours, cells were washed with PBS and fresh media, supplemented with 5% heat-inactivated human AB serum, was added. After 3 days, cells were co-infected with serum opsonised-*Cryptococcus* Kn99α-mCherry for 2 hours, washed to remove extracellular fungal cells, and replenished with fresh serum free-media.

### Live imaging

Infected samples were kept at 37°C with 5% CO_2_ in the imaging chamber of a Ti-E Nikon Epifluorescence microscope. Images were taken every 5 minutes over an 18-hour period and compiled into a single movie file using NIS Elements software. Movies were blinded by a third party before manual scoring for phagocytosis of *Cryptococcus*, virus infection rates, vomocytosis events, intracellular proliferation rates and macrophage integrity. See S1 Movie for an example vomocytosis event.

### Growth curve assay

A 10-fold diluted cryptococcal overnight culture was inoculated into YPD broth in a 48-well plate (final dilution in well: 1000-fold), in the presence or absence of type-I interferons. The plate was sealed with a breathable membrane and incubated at 37°C within a fully automated plate reader (FLUOStar, BMG Omega). Optical density readings at 600 nm were taken every 30 minutes over a 24 hour-period, with orbital shaking in between readings.

### Data analysis

Statistical analysis was performed using GraphPad Prism 6. Categorical data of phagocytosis or vomocytosis occurrence in the different conditions was assessed using Chi^2^ test and Fisher’s exact test. If data was normally distributed as assessed by Shapiro-Wilk test, then it was compared using Student’s t test. Figures show percentage of *Cryptococcus*-infected macrophages experiencing at least one vomocytosis event within each experiment. For intracellular proliferation rates, data was analysed using Mann-Whitney test. Growth curves were fitted to sigmoidal curves and the parameters were compared using Kruskal-Wallis test. All data shown corresponds to at least three independent experiments.

Raw data (collated manual counts for multiple time-lapse movies) are provided as supplemental material for each figure. Original time-lapse movies, upon which manual scoring was performed, are freely available upon request from the authors.

## Supporting information

S1 Fig **A.** Human monocyte-derived macrophages were infected with VLPs as indicated. After 24 hours, viral infection was assessed by p24 staining (blue). **B.** Cells were infected with VLPs as indicated, and subsequently infected with *C*. *neoformans*. Time-lapse microscopy videos were manually scored for vomocytosis. Graph shows percentage of *Cryptococcus*-infected macrophages which have experienced at least one vomocytosis event. Chi^2^ test followed by Fisher's exact test performed on raw vomocytosis counts from 5 independent experiments.
(TIFF)

S2 Fig Human monocyte-derived macrophages were infected with C. neoformans and stimulated with polyIC, in the presence of TBK1:IKKε inhibitor or DMSO control. **A.** Time-lapse microscopy videos were manually scored for vomocytosis. The graph shows the effect of each treatment as the percentage of *Cryptococcus*-infected macrophages which have experienced at least one vomocytosis event. Pooled data from 2 independent experiments. **B.** To check efficacy of the TBK1:IKKε inhibitor, levels of IFNα present in the culture supernatant were analysed by ELISA. The graph shows production of IFNα as fold change with respect to polyIC + DMSO stimulation. Significance represents one-sample T-test versus normalized value of 1.0.
(TIFF)

S3 Fig **A-B.** Cryptoccocal cells were grown in the presence or absence of IFNα (A) or IFNβ (B) over 24 hours. Growth was assessed by optical density readings at 600 nm. **C-F.** Human monocyte-derived macrophages were infected with *C*. *neoformans* in the presence of different doses of recombinant IFNα or IFNβ. Time-lapse microscopy videos were manually scored for phagocytosis (C and D) and intracellular proliferation rate of the fungus (E and F). Pooled data from 3 independent experiments.
(TIFF)

S1 Movie AVI movie showing vomocytosis event from a measles-infected monocyte-derived macrophage (green cell, left of image). Note that whilst some intracellular Cryptococci are expelled, others are retained, as is the intracellular *Cryptococcus* in the neighbouring cell (which is not measles-infected). Frames are 5 minutes apart in real time. Movie was captured using NIS Elements and then cropped and brightness enhanced for clarity in FIJI.
(AVI)

## References

[1] Isaacs A, Lindenmann J. Virus interference. I. The interferon. Proc R Soc Lond B Biol Sci. 1957;147(927):258–67. 10.1098/rspb.1957.0048 13465720

[2] Haller O, Arnheiter H, Gresser I, Lindenmann J. Virus-specific interferon action. Protection of newborn Mx carriers against lethal infection with influenza virus. J Exp Med. 1981;154(1):199–203. 10.1084/jem.154.1.199 6166723 PMC2186399

[3] Muller U, Steinhoff U, Reis LF, Hemmi S, Pavlovic J, Zinkernagel RM, et al Functional role of type I and type II interferons in antiviral defense. Science. 1994;264(5167):1918–21. 10.1126/science.8009221 8009221

[4] Yan N, Chen ZJ. Intrinsic antiviral immunity. Nat Immunol. 2012;13(3):214–22. 10.1038/ni.2229 22344284 PMC3549670

[5] MacMicking JD. Interferon-inducible effector mechanisms in cell-autonomous immunity. Nat Rev Immunol. 2012;12(5):367–82. 10.1038/nri3210 22531325 PMC4150610

[6] Mancuso G, Midiri A, Biondo C, Beninati C, Zummo S, Galbo R, et al Type I IFN signaling is crucial for host resistance against different species of pathogenic bacteria. J Immunol. 2007;178(5):3126–33. 10.4049/jimmunol.178.5.3126 17312160

[7] Bergstrom B, Aune MH, Awuh JA, Kojen JF, Blix KJ, Ryan L, et al TLR8 Senses Staphylococcus aureus RNA in Human Primary Monocytes and Macrophages and Induces IFN-beta Production via a TAK1-IKKbeta-IRF5 Signaling Pathway. J Immunol. 2015;195(3):1100–11. 10.4049/jimmunol.1403176 26085680

[8] Kagan JC, Su T, Horng T, Chow A, Akira S, Medzhitov R. TRAM couples endocytosis of Toll-like receptor 4 to the induction of interferon-beta. Nat Immunol. 2008;9(4):361–8. 10.1038/ni1569 18297073 PMC4112825

[9] Mancuso G, Gambuzza M, Midiri A, Biondo C, Papasergi S, Akira S, et al Bacterial recognition by TLR7 in the lysosomes of conventional dendritic cells. Nat Immunol. 2009;10(6):587–94. 10.1038/ni.1733 19430477

[10] Castiglia V, Piersigilli A, Ebner F, Janos M, Goldmann O, Dambock U, et al Type I Interferon Signaling Prevents IL-1beta-Driven Lethal Systemic Hyperinflammation during Invasive Bacterial Infection of Soft Tissue. Cell Host Microbe. 2016;19(3):375–87. 10.1016/j.chom.2016.02.003 26962946

[11] Gratz N, Hartweger H, Matt U, Kratochvill F, Janos M, Sigel S, et al Type I interferon production induced by Streptococcus pyogenes-derived nucleic acids is required for host protection. PLoS Pathog. 2011;7(5):e1001345 10.1371/journal.ppat.1001345 21625574 PMC3098218

[12] Webb LM, Lundie RJ, Borger JG, Brown SL, Connor LM, Cartwright AN, et al Type I interferon is required for T helper (Th) 2 induction by dendritic cells. EMBO J. 2017;36(16):2404–18. 10.15252/embj.201695345 28716804 PMC5556270

[13] Rajasingham R, Smith RM, Park BJ, Jarvis JN, Govender NP, Chiller TM, et al Global burden of disease of HIV-associated cryptococcal meningitis: an updated analysis. Lancet Infect Dis. 2017;17(8):873–81. 10.1016/S1473-3099(17)30243-8 28483415 PMC5818156

[14] Alvarez M, Casadevall A. Cell-to-cell spread and massive vacuole formation after Cryptococcus neoformans infection of murine macrophages. BMC Immunol. 2007;8:16 10.1186/1471-2172-8-16 17705844 PMC1988836

[15] Feldmesser M, Kress Y, Novikoff P, Casadevall A. Cryptococcus neoformans is a facultative intracellular pathogen in murine pulmonary infection. Infect Immun. 2000;68(7):4225–37. 10.1128/iai.68.7.4225-4237.2000 10858240 PMC101732

[16] Tucker SC, Casadevall A. Replication of Cryptococcus neoformans in macrophages is accompanied by phagosomal permeabilization and accumulation of vesicles containing polysaccharide in the cytoplasm. Proc Natl Acad Sci U S A. 2002;99(5):3165–70. 10.1073/pnas.052702799 11880650 PMC122490

[17] Voelz K, May RC. Cryptococcal interactions with the host immune system. Eukaryot Cell. 2010;9(6):835–46. 10.1128/EC.00039-10 20382758 PMC2901644

[18] Osterholzer JJ, Milam JE, Chen GH, Toews GB, Huffnagle GB, Olszewski MA. Role of dendritic cells and alveolar macrophages in regulating early host defense against pulmonary infection with Cryptococcus neoformans. Infect Immun. 2009;77(9):3749–58. 10.1128/IAI.00454-09 19564388 PMC2737986

[19] Sorrell TC, Juillard PG, Djordjevic JT, Kaufman-Francis K, Dietmann A, Milonig A, et al Cryptococcal transmigration across a model brain blood-barrier: evidence of the Trojan horse mechanism and differences between Cryptococcus neoformans var. grubii strain H99 and Cryptococcus gattii strain R265. Microbes Infect. 2016;18(1):57–67. 10.1016/j.micinf.2015.08.017 26369713

[20] Alvarez M, Casadevall A. Phagosome extrusion and host-cell survival after Cryptococcus neoformans phagocytosis by macrophages. Curr Biol. 2006;16(21):2161–5. 10.1016/j.cub.2006.09.061 17084702

[21] Ma H, Croudace JE, Lammas DA, May RC. Expulsion of live pathogenic yeast by macrophages. Curr Biol. 2006;16(21):2156–60. 10.1016/j.cub.2006.09.032 17084701

[22] Sionov E, Mayer-Barber KD, Chang YC, Kauffman KD, Eckhaus MA, Salazar AM, et al Type I IFN Induction via Poly-ICLC Protects Mice against Cryptococcosis. PLoS Pathog. 2015;11(8):e1005040 10.1371/journal.ppat.1005040 26252005 PMC4529209

[23] Goujon C, Arfi V, Pertel T, Luban J, Lienard J, Rigal D, et al Characterization of simian immunodeficiency virus SIVSM/human immunodeficiency virus type 2 Vpx function in human myeloid cells. J Virol. 2008;82(24):12335–45. 10.1128/JVI.01181-08 18829761 PMC2593360

[24] Mlcochova P, Watters SA, Towers GJ, Noursadeghi M, Gupta RK. Vpx complementation of 'non-macrophage tropic' R5 viruses reveals robust entry of infectious HIV-1 cores into macrophages. Retrovirology. 2014;11:25 10.1186/1742-4690-11-25 24656066 PMC3997928

[25] Allen IV, McQuaid S, Penalva R, Ludlow M, Duprex WP, Rima BK. Macrophages and Dendritic Cells Are the Predominant Cells Infected in Measles in Humans. mSphere. 2018;3(3). 10.1128/mSphere.00570-17 PMC5956143 29743202

[26] Garcia-Rodas R, Gonzalez-Camacho F, Rodriguez-Tudela JL, Cuenca-Estrella M, Zaragoza O. The interaction between Candida krusei and murine macrophages results in multiple outcomes, including intracellular survival and escape from killing. Infect Immun. 2011;79(6):2136–44. 10.1128/IAI.00044-11 21422181 PMC3125833

[27] Bain JM, Lewis LE, Okai B, Quinn J, Gow NA, Erwig LP. Non-lytic expulsion/exocytosis of Candida albicans from macrophages. Fungal Genet Biol. 2012;49(9):677–8. 10.1016/j.fgb.2012.01.008 22326419 PMC3430864

[28] Braun BR, Kadosh D, Johnson AD. NRG1, a repressor of filamentous growth in C.albicans, is down-regulated during filament induction. EMBO J. 2001;20(17):4753–61. 10.1093/emboj/20.17.4753 11532939 PMC125265

[29] Alexopoulou L, Holt AC, Medzhitov R, Flavell RA. Recognition of double-stranded RNA and activation of NF-kappaB by Toll-like receptor 3. Nature. 2001;413(6857):732–8. 10.1038/35099560 11607032

[30] Hardy GA, Sieg S, Rodriguez B, Anthony D, Asaad R, Jiang W, et al Interferon-alpha is the primary plasma type-I IFN in HIV-1 infection and correlates with immune activation and disease markers. PLoS One. 2013;8(2):e56527 10.1371/journal.pone.0056527 23437155 PMC3577907

[31] Rasaiyaah J, Tan CP, Fletcher AJ, Price AJ, Blondeau C, Hilditch L, et al HIV-1 evades innate immune recognition through specific cofactor recruitment. Nature. 2013;503(7476):402–5. 10.1038/nature12769 24196705 PMC3928559

[32] Tsang J, Chain BM, Miller RF, Webb BL, Barclay W, Towers GJ, et al HIV-1 infection of macrophages is dependent on evasion of innate immune cellular activation. AIDS. 2009;23(17):2255–63. 10.1097/QAD.0b013e328331a4ce 19741482 PMC2873676

[33] Sato K, Yamamoto H, Nomura T, Matsumoto I, Miyasaka T, Zong T, et al Cryptococcus neoformans Infection in Mice Lacking Type I Interferon Signaling Leads to Increased Fungal Clearance and IL-4-Dependent Mucin Production in the Lungs. PLoS One. 2015;10(9):e0138291 10.1371/journal.pone.0138291 26384031 PMC4575107

[34] Oliveira LVN, Costa MC, Magalhaes TFF, Bastos RW, Santos PC, Carneiro HCS, et al Influenza A Virus as a Predisposing Factor for Cryptococcosis. Front Cell Infect Microbiol. 2017;7:419 10.3389/fcimb.2017.00419 29018774 PMC5622999

[35] Garelnabi M, Taylor-Smith LM, Bielska E, Hall RA, Stones D, May RC. Quantifying donor-to-donor variation in macrophage responses to the human fungal pathogen Cryptococcus neoformans. PLoS One. 2018;13(3):e0194615 10.1371/journal.pone.0194615 29596441 PMC5875765

[36] Upadhya R, Lam WC, Maybruck BT, Donlin MJ, Chang AL, Kayode S, et al A fluorogenic C. neoformans reporter strain with a robust expression of m-cherry expressed from a safe haven site in the genome. Fungal Genet Biol. 2017;108:13–25. 10.1016/j.fgb.2017.08.008 28870457 PMC5681388

[37] Goujon C, Jarrosson-Wuilleme L, Bernaud J, Rigal D, Darlix JL, Cimarelli A. With a little help from a friend: increasing HIV transduction of monocyte-derived dendritic cells with virion-like particles of SIV(MAC). Gene Ther. 2006;13(12):991–4. 10.1038/sj.gt.3302753 16525481

[38] Hashimoto K, Ono N, Tatsuo H, Minagawa H, Takeda M, Takeuchi K, et al SLAM (CD150)-independent measles virus entry as revealed by recombinant virus expressing green fluorescent protein. J Virol. 2002;76(13):6743–9. 10.1128/JVI.76.13.6743-6749.2002 12050387 PMC136249

[39] Takeda M, Takeuchi K, Miyajima N, Kobune F, Ami Y, Nagata N, et al Recovery of pathogenic measles virus from cloned cDNA. J Virol. 2000;74(14):6643–7. 10.1128/jvi.74.14.6643-6647.2000 10864679 PMC112175

