# Supplementary figures and images for: Viral infection triggers interferon-induced expulsion of live Cryptococcus neoformans by macrophages
Source: PLoS Pathog. 2020 Feb 27;16(2):e1008240. doi: 10.1371/journal.ppat.1008240 (PMC7046190; doi:10.1371/journal.ppat.1008240)

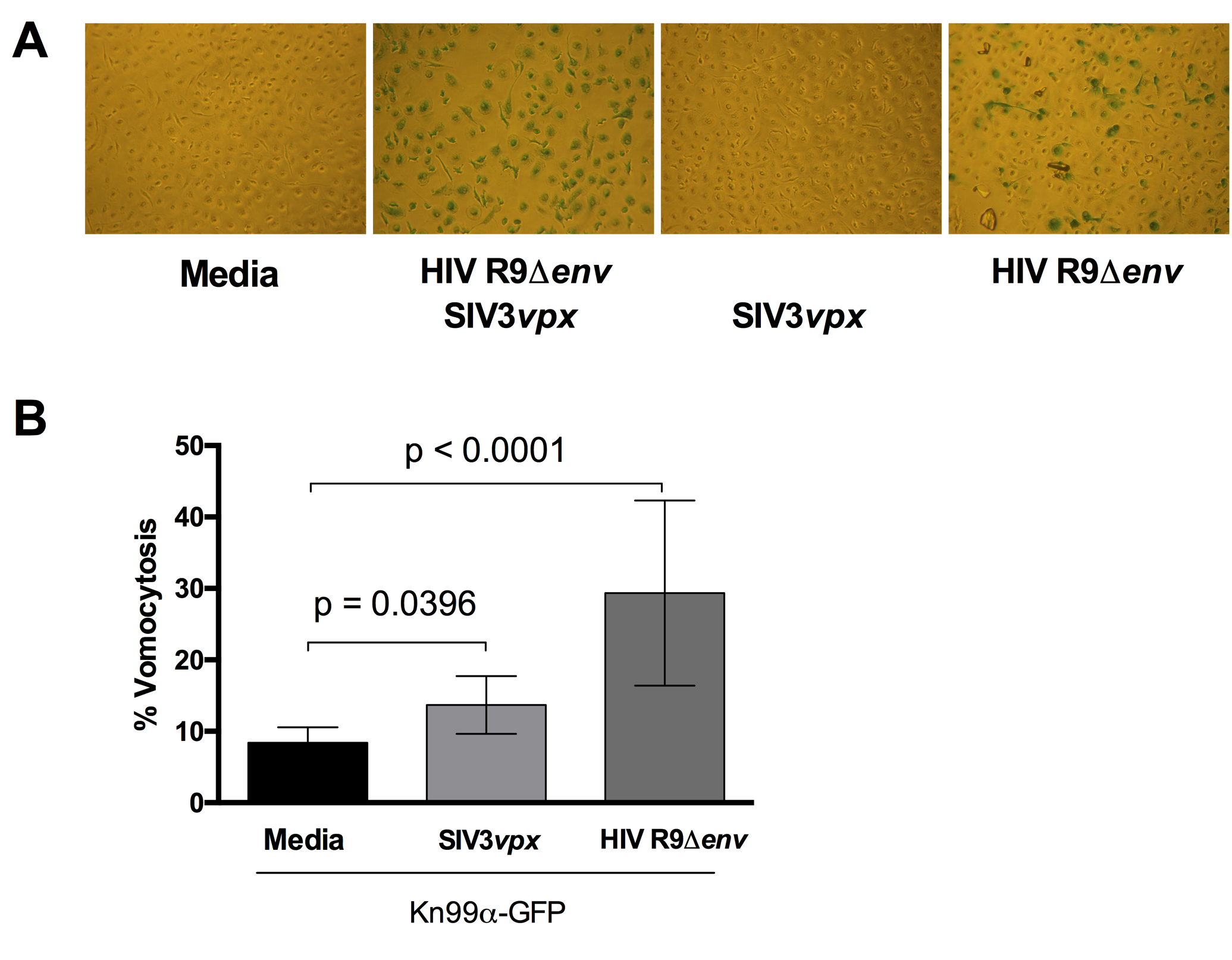

Supplement: S1 Fig — A. Human monocyte-derived macrophages were infected with VLPs as indicated. After 24 hours, viral infection was assessed by p24 staining (blue). B. Cells were infected with VLPs as indicated, and subsequently infected with C. neoformans. Time-lapse microscopy videos were manually scored for vomocytosis. Graph shows percentage of Cryptococcus-infected macrophages which have experienced at least one vomocytosis event. Chi2 test followed by Fisher's exact test performed on raw vomocytosis counts from 5 independent experiments. (TIFF) [file ppat.1008240.s001.tiff]

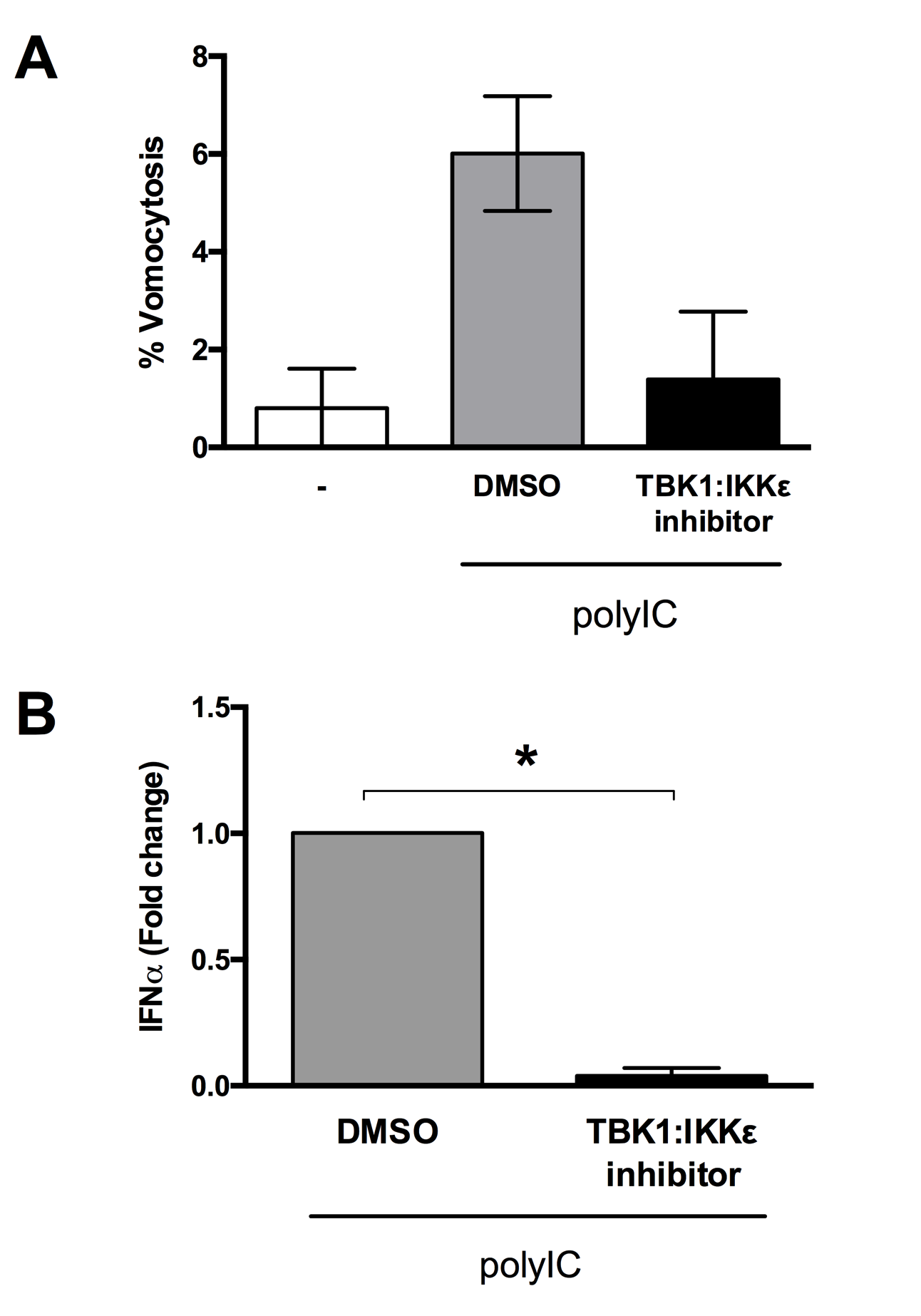

Supplement: S2 Fig — A. Time-lapse microscopy videos were manually scored for vomocytosis. The graph shows the effect of each treatment as the percentage of Cryptococcus-infected macrophages which have experienced at least one vomocytosis event. Pooled data from 2 independent experiments. B. To check efficacy of the TBK1:IKKε inhibitor, levels of IFNα present in the culture supernatant were analysed by ELISA. The graph shows production of IFNα as fold change with respect to polyIC + DMSO stimulation. Significance represents one-sample T-test versus normalized value of 1.0. (TIFF) [file ppat.1008240.s002.tiff]

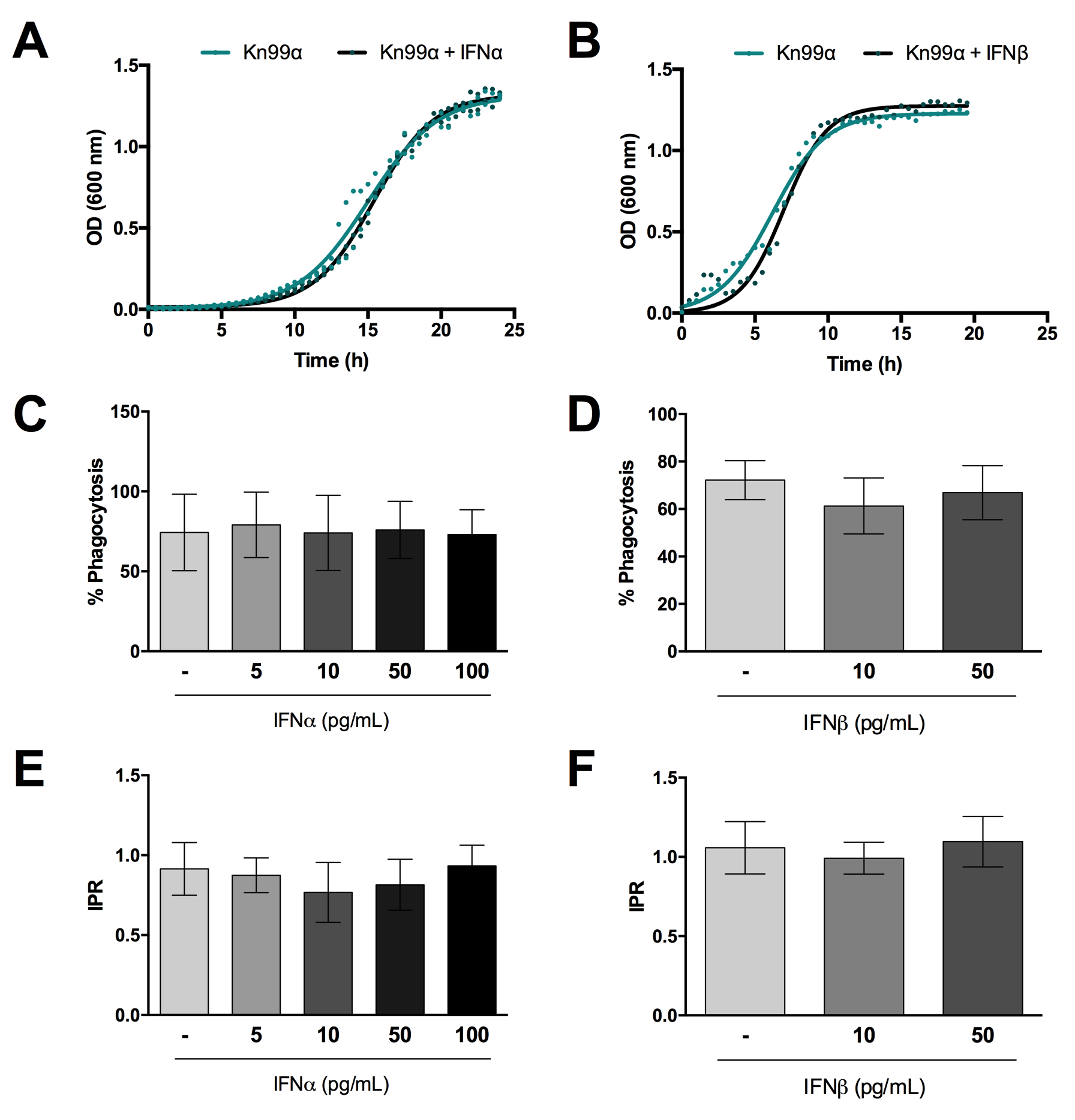

Supplement: S3 Fig — A-B. Cryptoccocal cells were grown in the presence or absence of IFNα (A) or IFNβ (B) over 24 hours. Growth was assessed by optical density readings at 600 nm. C-F. Human monocyte-derived macrophages were infected with C. neoformans in the presence of different doses of recombinant IFNα or IFNβ. Time-lapse microscopy videos were manually scored for phagocytosis (C and D) and intracellular proliferation rate of the fungus (E and F). Pooled data from 3 independent experiments. (TIFF) [file ppat.1008240.s003.tiff]
